# Implementing the CRISPR 2.1 array for genotyping enhances identification of uropathogenic *Escherichia coli* in Colombian pediatric patients

**DOI:** 10.7705/biomedica.7743

**Published:** 2026-04-28

**Authors:** Angélica Ramírez, Ricardo Aislant, Santiago Quevedo, Andrea Sarmiento, Lilian Dulcey, Laura Vargas, Mayra Machucas, José Arturo Gutiérrez-Triana

**Affiliations:** 1 Grupo de Inmunología y Epidemiología Molecular, Escuela de Microbiología, Facultad de Salud, Universidad Industrial de Santander, Bucaramanga, Colombia Universidad Industrial de Santander Grupo de Inmunología y Epidemiología Molecular Escuela de Microbiología, Facultad de Salud Universidad Industrial de Santander Bucaramanga Colombia

**Keywords:** Uropathogenic *Escherichia coli*, molecular typing, drug resistance., *Escherichia coli* uropatógena, tipificación molecular, resistencia a medicamentos

## Abstract

**Introduction.:**

The emergence of antibiotic resistance in *Escherichia coli* has contributed to the increasing incidence and lethality of urinary tract infections.

**Objective.:**

To characterize uropathogenic *E. coli* isolates obtained from patients with urinary tract infections in two hospitals in Bucaramanga, Colombia. Specifically, we assessed the significance of CRISPR 2.1 *locus* elements as a target for genotyping.

**Materials and methods.:**

We combined phylotyping based on the Clermont classification, antimicrobial susceptibility testing approaches, and PCR amplification of the CRISPR 2.1 array.

**Results.:**

We found that the variation in the CRISPR 2.1 array, along with the presence of resistance and virulence genes, phylogroups, and antibiotic sensitivity patterns provide significant and reliable data for epidemiological studies. The analysis of 72 isolates revealed considerable resistance to ampicillin and trimethoprim/sulfamethoxazole, with rates of 80% and 60%, respectively. The isolates also demonstrated the occurrence of *bla*_*CTX-M”*_ *bla*_*TEM”*_ and *bla*_*SHV*_ genes in 54.55, 18.18, and 4.17% of the cases, respectively. According to the Clermont classification, groups D and B2 accounted for 65.2% of all isolates. The CRISPR 2.1 array was present in most isolates except those in group B2 and exhibited length polymorphism.

**Conclusions.:**

Our results emphasized the utility of CRISPR 2.1 array polymorphism in reliably identifying uropathogenic *E. coli* and enhancing distinction, which is a noteworthy advance in regional epidemiological monitoring systems.

## Resumen

**Introducción.:**

La aparición de resistencia a los antibióticos en *Escherichia coli* ha contribuido a la creciente incidencia y letalidad de las infecciones urinarias.

**Objetivos.:**

Caracterizar aislamientos de cepas uropatógenas de *E. coli* obtenidos de pacientes con infecciones urinarias de dos hospitales de Bucaramanga, Colombia. Se evaluó específicamente la relevancia del *locus* CRISPR 2.1 como elemento informativo para la genotipificación.

**Materiales y métodos.:**

Se combinó la filotipificación basada en la clasificación de Clermont y los antibiogramas, junto con la amplificación por PCR del arreglo CRIPSR 2.1.

**Resultados.:**

Se encontró que la variación del arreglo CRISPR 2.1, así como la presencia de genes de resistencia y virulencia, filogrupos y patrones de sensibilidad a los antibióticos, proporcionan datos significativos y confiables para los estudios epidemiológicos. El análisis de 72 aislamientos reveló una resistencia significativa a la ampicilina y al trimetoprim sulfametoxazol, con tasas del 80 y el 60 %, respectivamente. Además, los aislamientos demostraron la presencia de los genes *bla*_*CTX-M”*_ *bla*_*TEM*_ *”* y *bla*_*SHV*_ en el 54,55, 18,18 y 4,17 % de los casos, respectivamente. Los grupos D y B2 comprendieron el 65,2 % de los aislamientos según la clasificación de Clermont. El arreglo CRISPR 2.1 se encontró en la mayoría de los aislamientos, excepto en los del grupo B2, y exhibió polimorfismo de longitud.

**Conclusiones.:**

Los resultados enfatizan la utilidad del polimorfismo presentado por el arreglo CRISPR 2.1 en la subtipificación de las cepas de *E. coli* uropatógenas, lo que contribuye a mejorar la identificación bacteriana y supone un avance notable en el establecimiento de sistemas regionales de vigilancia epidemiológica.

Urinary tract infections are prevalent bacterial infections that can lead to substantial medical expenses for hospitalized patients, rather than severe health complications or potential mortality ^1^^,^^2^. *Escherichia coli*, a Gramnegative bacterium commonly found in the normal gastrointestinal microbiota, is responsible for around 75% of these types of infections. Virulence factors, as different types of adhesins, fimbriae, and exotoxins, significantly contribute to the ability of uropathogenic *E. coli* strains to cause disease ^3^. Furthermore, the growing prevalence of antibiotic-resistant uropathogenic *E. coli* strains is a significant threat to public health, with variations in resistance patterns observed across different regions and periods. Besides, certain bacteria possess intrinsic resistance to antibiotics ^4^.

*Escherichia coli* can also transfer resistance genes horizontally through mobile genetic elements like pathogenic islands, plasmids, transposons, and integrons, which facilitate the dissemination of genes, such as those that encode extended-spectrum beta-lactamase genes. These genes confer resistance to several classes of p-lactam antibiotics like penicillins, monobactams, and third-generation cephalosporins, contributing to the emergence and worldwide spread of multidrug-resistant *E. coli* strains that have rendered many antibiotics ineffective ^5^^,^^6^.

To fully understand the uropathogenic *E coli* strains is crucial for testing antibiotic resistance, confirming the existence of resistance genes, and evaluating the genotypes of the strains in circulation. It is also essential to trace the evolution of these bacteria over time and gain a thorough understanding of their pathogenicity mechanism and antibiotic resistance ^3^^,^^7^^-^^9^.

Several typing methods have been developed to characterize bacterial isolates, from traditional phenotypic typing to advanced approaches that examine the genetic composition of bacteria. These modern methods have proven highly effective in microbial typing. The most commonly used methods include pulsed-field gel electrophoresis (PFGE) ^10^, analysis of plasmid profiles ^11^, the Clermont method (phylotyping) ^12^, restriction fragment length polymorphism (RFLP) ^13^^,^^14^, ribotyping ^15^, multilocus sequence typing (MLST) ^10^^,^^16^, whole-genome sequencing (WGS) ^17^, repetitive extragenic palindromic (REO) PCR ^10^, and enterobacterial repetitive intergenic consensus (ERIC) PCR ^18^^,^^19^.

The WGS method Is recognized as the gold standard for identifying strains, while MLST is widely used in practice. Unfortunately, both methods can be expensive and inaccessible to many laboratories ^20^^,^^21^.

Recently, the CRISPR-Cas *locus* has been used in the genotyping of pathogenic microorganisms ^22^^,^^23^. CRISPR, an acronym for clustered regularly interspaced short palindromic repeats, is a mechanism that allows prokaryotes to adaptively defend against invading DNA or RNA molecules ^24^^,^^25^.

The CRISPR system is classified into two classes (1 and 2), six types (I to VI), and several subtypes, depending on the diversity of Cas genes and the characteristics of the interference complex ^26^. There are two distinct CRISPR-Cas subtypes in *E. coli*: I-E and I-F. For subtype I-E, the three cassettes are CRISPR 2.1, 2.2, and 2.3. Among these, the CRISPR 2.1 *locus* has the highest degree of polymorphism ^23^^,^^27^^-^^29^.

Here, we analyzed *E. coli* samples isolated from pediatric patients with urinary tract infections at hospitals in Bucaramanga, Colombia. We used both phenotypic and genotypic techniques to evaluate these clinical isolates. We specifically assessed the antibiotic sensitivity pattern, resistance and virulence genes, phylogroup, and the polymorphism of the CRISPR 2.1 array. The resulting data provide novel insights into the diversity analysis of the CRISPR 2.1 array in *E. coli*, which highlights its significance as a genotyping tool and its relationship with genes linked to resistance and virulence.

## Materials and methods

We collected 72 *E. coli* isolates from pediatric patients with urinary tract infections treated at two hospitals in Bucaramanga, Colombia, from August 2021 to May 2022. The bacterial isolates were identified as *E. coli* using the BD Phoenix™ automated identification and susceptibility testing system and then cryopreserved at -70°C in BHI broth with 20% glycerol.

### Antimicrobial susceptibility testing

We assessed the antimicrobial susceptibility profile on Mueller-Hinton agar plates using the Kirby-Bauer disk diffusion method and following the guidelines of the Clinical and Laboratory Standards Institute (CLSI) (30). We tested 14 antibiotics: cefoxitin (30 µg), cefotaxime (30 µg), ceftriaxone (30 µg), ceftazidime (30 µg), piperacillin-tazobactam (110 µg), ampicillin (10 µg), ertapenem (10 µg), imipenem (10 µg), meropenem (10 µg), amikacin (30 µg), gentamicin (120 µg), levofloxacin (5 µg), nitrofurantoin (300 µg), and trimethoprim-sulfamethoxazole (25 µg) (appendix 1). We determined antibiotic resistance according to the breakpoint proposed by CLSI ^30^. For quality control, we used *E. coli* ATCC™25922™

### Phylotyping and detection of ESBL (Extended-Spectrum fi-Lactamases) genes

We isolated the genomic DNA of *E. coli* strains using the salting out method ^31^ from cultures grown overnight in BHI broth at 37 °C. Briefly, the bacterial pellet was resuspended in ultrapure water with 1 mg/ml of lysozyme, 0.56 mg/ml of proteinase K, and 1% of sodium dodecyl sulfate, incubated at 56 °C for 30 minutes, heated to 100 °C for 15 minutes, and then alternately incubated every 5 minutes with mechanical stirring to promote bacterial lysis. The proteins were precipitated by adding 5M NaCI (to a final concentration of 1.7M) and centrifuged at 15 000 rpm, for 5 minutes. DNA was precipitated at -20 °C with isopropanol, centrifuged at 15 000 rpm, rinsed with 70% ethanol, air-dried, resuspended in 1XTE buffer, and quantified using a spectrophotometer.

We confirmed *E. coli* in all clinical isolates by amplifying a specific portion of the 16S rRNA gene that is unique to these bacteria, using a previously reported method (table 1) ^32^^-^^34^. Pylotyping was done according to the protocol established by Clermont *et al*. ^12^. Briefly, a multiplex PCR was used to detect *chuA, yjaA*, TspE4C2, and *arpA* genes, and identify the four major phylogenetic groups: A, B1, D, and B2 (table 2). Additional PCR amplifications were performed to detect a different region of the *arpA* gene and the *trpA* gene to identify E and C groups (table 2). The amplification conditions for the multiplex PCR, *trpA* and *arpA* genes, and the sequences of the primers used are shown in table 1.

**Table 1.** Primers and PCR conditions

PCR	Gene	Primer	Primer sequences	Amplified product (Bp)	Amplification conditions	References
16S rRNA	gene identification					
16S rRNA	*16S rRNA*	ECO-1	5'-GACCTCGGTTTAGTTCACAGA-3'	585	Denaturation: 95°C x 2 min. Then 95°C x 20 s, 60°C x 30 s, 72°C x 30 s for 30 cycles. Extension: 72°C x 5 min.	^38^
		ECO-2	5'-CACACGCTGACGCTGACCA-3'			
Resistance determinants						
B-lactam antibiotic resistance	*blaCTX-M*	CTXM_F	5'-GACGATGTCACTGGCTGAGCTTAGC-3'	499	Denaturation: 94°C x 4 min. Then 94°C x 1 min, 59°C x 10 s, 72°C x 30 s for 30 cycles. Extension: 72°C x 5 min.	^39^
		CTXM_R	5'-AGCCGCCGACGCTAATACA-3'			
	*blaTEM*	TEM_F	5'-TTCTTGAAGACGAAAGGGC-3'	1150	Denaturation: 94°C x 4 min. Then 94°C x 1 min, 61°C x 30 s, 72°C x 1 min for 30 cycles. Extension: 72°C x 5 min.	^40^
		TEM_R	5'-ACGCTCAGTGGAACGAAAAC-3'			
	*blaSHV*	SHV_F	5'-GATGAACGCTTTCCCATGATG-3'	214	Denaturation: 94°C x 4 min. Then 94°C x 1 min, 59°C x 30 s, 72°C x 1 min for 30 cycles. Extension: 72°C x 5 min.	^39^
			5'-CGCTGTTATCGCTCATGGTAA-3'			
CRISPR 2.1 array						
CRISPR 2.1	*iap*	iap_F	5'-TGGTGAAGGAGTTGGCGAAGG-3'	500-2000	Denaturation: 94°C x 30 s. Then 94°C x 15 s, 68°C x 1 min, 68°C x 2 min for 10 cycles. Then 94°C x 15 s, 65°C x 1 min, 68°C x 2 min for 10 cycles. Then 94°C x 15 s, 60°C x 1 min, 68°C x 2 min for 15 cycles. Final extension: 68°C x 5 min.	^32^
	*cas2*	Cas2_R	5'-AAAATGTCCCTCCGCGCTTACG-3'			
Determination of phylogenetic groups according to Clermont						
Multiplex PCR	*chuA*	chuA_F	5'-ATGGTACCGGACGAACCAAC-3'	288	Denaturation: 94°C x 4 min. Then 94°C x 30 s, 59°C x 30 s, 72°C x 30 s for 30 cycles. Extension: 72°C x 5 min.	^20^
		chuA_R	5'-TGCCGCCAGTACCAAAGACA-3'			
	*yjaA*	yjaA_F	5'-CAAACGTGAAGTGTCAGGAG-3'	211		
		yjaA_R	5'-AATGCGTTCCTCAACCTGTG-3'			
	*TspE4.C2*	TspE4C2_F	5'-CACTATTCGTAAGGTCATCC-3'	152		
		TspE4C2_R	5'-AGTTTATCGCTGCGGGTCGC-3'			
	*arpA*	AceK_F	5'-AACGCTATTCGCCAGCTTGC-3'	400		
		ArpA1_R	5'-TCTCCCCATACCGTACGCTA-3'			
Group E	*arpA*	ArpAgpE_F	5'-GATTCCATCTTGTCAAAATATGCC-3'	301	Denaturation: 94°C x 4 min. Then 94°C x 30 s, 57°C x 30 s, 72°C x 30 s for 30 cycles. Extension: 72°C x 45 s.	
		ArpAgp_R	5'-GAAAAGAAAAAGAATTCCCAAGAG-3'			
Group C	*trpA*	trpAgpC_F	5'-AGTTTTATGCCCAGTGCGAG-3'	219	Denaturation: 94°C x 4 min. Then 94°C x 30 s, 59°C x 30 s, 72°C x 30 s for 30 cycles. Extension: 72°C x 5 min.	
		trpAgpC_R	5'-TCTGCGCCGGTCACGCCC-3'			
Internal control	*trpA*	trpBA_F	5'-CGGCGATAAAGACATCTTCAC-3'	489		
		trpBA_R	5'-GCAACGCGGCCTGGCGGAAG-3'			

16S rRNA: 16S ribosomal ribonucleic acid; blaCTX-M: beta-lactamase CTX-M; blaSHV: beta-lactamase SHV; blaTEM: beta-lactamase TEM; Bp: base pairs; cas2: CRISPR associated protein 2; CRISPR: clustered regularly interspaced short palindromic repeats; iap: invasion-associated protein gene; rRNA: ribosomal ribonucleic acid; trpA: tryptophan synthase subunit A gene

**Table 2:** Amplification profiles used to assign phylogenetic groups based on Clermont's method

Genotype				Phylogenetic
***arpA* (400 bp)**	***chuA* (288 bp)**	***yjaA*( 211 bp)**	***TspE4C2* (152 bp)**	group
+	-	-	-	A
+	-	-	+	B1
-	+	-	-	F
-	+	+	-	B2
-	+	+	+	B2
-	+	-	+	B2
+	-	+	-	A/C**
+	+	-	-	D/E***
+	+	-	+	D/E***
+	+	+	-	E or Clade 1
-	-	+	-	Clade 1 or II
-	476 bp*	-	-	Clade III, IV or V
-	-	-	+	Undetermined
-	-	+	+	Undetermined
+	-	+	+	Undetermined
+	+	+	+	Undetermined
-	-	-	-	Undetermined

arpA: acyl carrier protein gene A; bp: base pairs; PCR: Polymerase Chain Reaction; TspE4C2: TspE4 insertion element C2

* Amplicon is obtained only in isolates belonging to clades III, IV or V.

** Confirm through specific primers for group C’s PCR. If an amplicon is obtained, the isolate belongs to group C, if not, it belongs to A.

*** Confirm through specific primers for group E’s PCR. If an amplicon is obtained, the isolate belongs to group E, if not, it belongs to D.

PCR was used to assess the presence of the ESBL (Extended-Spectrum β-Lactamases) genes *bla*_*CTX-M”*_ *bla*_*TEM”*_ , and *bla*_*SHV”*_ . For the amplification reactions, we used 10 µM of each primer, 0.2 µM of dNTPs (deoxynucleotide), IX Taq Buffer, 0.25 U of Taq DNA Polymerase, and 7.5 ng/pl of DNA, as previously reported ^35^^-^^38^. The PCR amplification products were analyzed by electrophoresis in a 2% agarose gel. Primer sequences and amplification conditions are specified in table 1.

### CRISPR 2.1 array PCR amplification

The CRISPR 2.1 array was amplified by PCR using the primers previously described ^28^ (table 1). The PCR reaction consisted of 0.2 µM of each primer, 0.2 µM of dNTPs, 1X of b uffer OneTaq, 0.5 U of OneTaq polymerase (New England Labs, USA), and five ng/µl of DNA. The PCR conditions are described in table 1.

## Results

### Phylotyping based on the Clermont classification

Seventy-one of the 72 isolates were classified into one of the seven phylogenetic groups in table 3. The predominant phylogenetic groups observed were D (31.9%) and B2 (31.9%), collectively accounting for 65.2% of the isolates examined. However, one isolate could not be categorized into any phylogenetic groupings because its gene amplification pattern did not match any of the established patterns described by Clermont *et al.*^12^.

**Table 3.** Distribution of Escherichia coli isolates according to the Clermont classification

Phylogenetic groups	***E. coli* isolates**	
	n	%
A	7	9.7
B1	7	9.7
B2	23	31.9
C	6	8.3
D	24	33.3
E	2	2.8
F	2	2.8
Undetermined	1	1.4
Total	72	100

### Antibiotic resistance in Escherichia coli isolates

**Figure 1.**
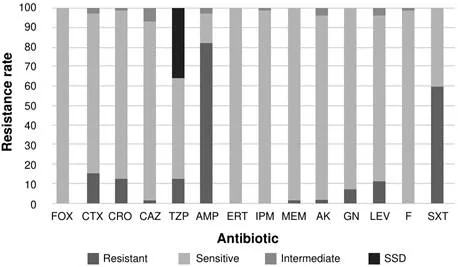
Resistance rates of the uropathogenic *Escherichia coli* isolates to a set of antibiotics

AK: amikacin; AMP: ampicillin; CAZ: ceftazidime; CRO: ceftriaxone; CTX: cefotaxime; ERT: ertapenem; F: nitrofurantoin; FOX: cefoxitin; GN: gentamicin; IPM: imipenem; LEV: levofloxacin; MEM: meropenem; SXT: sulfamethoxazole/trimethoprim; TZP: tazobactam-piperacillin; SDD: susceptible-dose dependent

Of the 14 antibiotics evaluated, ampicillin exhibited a resistance rate of 81.94% (n = 59), while for trimethoprim/sulfamethoxazole it was 59.72% (n = 43); ceftriaxone accounted for 12.5% and cefotaxime for 15.27%. All isolates were 100% sensitive to ertapenem and cefoxitin (figure 1).

The 11 isolates resistant to third-generation cephalosporins, specifically cefotaxime, ceftriaxone, and ceftazidime, were tested using the double-disk diffusion synergy test and a phenotypic assessment of antibiotic resistance. Using PCR, we determined the specific ESBL type of each of these 11 isolates. The resistance genes were detected in 81.82% (9/11) of the isolates, with the *bla*_*CTX-M*_ gene being the most common, present in 54.55% (6/11) of the isolates. Gene *bla*_*TEM*_ was detected in 18.18% (2/11) of the isolates, while *bla*_*SHV*_ was present in 9.09% (1/11). 

ESBL resistance gene screening revealed that 30 of 72 isolates included *bla*_*CTX-M*_ , *bla*_*TEM*_ , and *bla*_*SHV*_ . The highest prevalence corresponded to 29.17% of the isolates expressing *bla*_*TEM*_ (21/72), followed by *bla*_*CTX*_ -M in 8.33% (6/72), and *bla*_*SHV*_ in 4.17% (3/72).

### Implementing genotyping through the CRISPR 2.1 array

**Figure 2.**
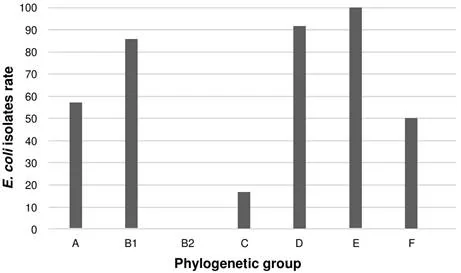
Distribution of the CRISPR 2.1 loci among different phylogenetic groups

**Figure 3.**
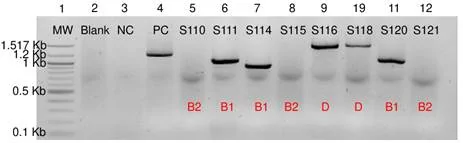
Amplicon length polymorphism of the CRISPR 2.1 array

Of the 72 *E. coli* isolates analyzed, 36 (50%) exhibited amplification of the CRISPR 2.1 array. This *locus* distribution varied among the Clermont phylogenetic groups: it was present in the majority of isolates belonging to groups A, B1, C, D, E, and F, but was completely absent in all group B2 isolates. Notably, all isolates classified as group E harbored the CRISPR 2.1 *locus*, while none of the B2 isolates did, indicating a striking divergence between these two major phylogenetic lineages (figure 2). Additionally, the CRISPR 2.1 *locus* displayed clear length polymorphisms among positive isolates, suggesting variability in spacer composition and potential value for subtyping (figure 3).

CRISPR: clustered regularly interspaced short palindromic repeats

CRISPR: clustered regularly interspaced short palindromic repeats

Agarose gel electrophoresis showing different CRISPR 2.1 amplicons from uropathogenic *Escherichia coli bacteria*.

1. MW. 2. Blank. 3. Negative control (NC) ATCC 25922 *E. coli*. 4. Positive control (PC) K12 *E. coli*. 5-12.

Uropathogenic *Escherichia coli* isolates. The phylogroup assigned to each isolate is shown in red.

To explore the interaction between adaptive immunity and antibiotic resistance, we examined the relationship between CRISPR 2.1 presence and the distribution of ESBL-type resistance genes (table 4). Interestingly, isolates with and without CRISPR 2.1 carried all three resistance genes, *bla*_*CTXM”*_ *bla*_*TEM”*_ and *bla*_*SHV*_ *”* in comparable proportions. This broad overlap suggests that the CRISPR 2.1 *locus* does not function as a barrier to the acquisition of these resistance determinants in our clinical strains. Although both CRISPR- positive and CRISPR-negative isolates harbored *bla*_*CTXM”*_ *bla*_*TEM”*_ and *bla*_*SHV”*_ genes, the statistical analysis revealed no significant association between CRISPR 2.1 status and the presence of any ESBL gene (Fisher’s exact test, p > 0.05). These results reinforce the idea that, at least in *E. coli*, CRISPR-based defense, may not effectively counteract horizontal gene transfer of antibiotic resistance elements.

**Table 4.** Association of ESBLS-type resistance genes and CRISPR 2.1 in uropathogenic Escherichia coli *

Gene	CRISPR/Cas + (n = 36)	CRISPR/Cas - (n = 36)	P
*bla_CTX-M*	3 (8.3%)	3 (8.3%)	1
*bla_TEM*	10 (27.8%)	11 (30.6%)	1
*bla_SHV*	2 (5.6%)	1 (2.8%)	1

Cas: CRISPR-associated protein; CRISPR: clustered regularly interspaced short palindromic repeats; ESBL: extended-spectrum beta-lactamases

* Fisher's exact test (p < 0.05) was considered statistically significant.

## Discussion

Our study revealed a high level of resistance to various classes of antibiotics among the isolated uropathogenic *E. coli* strains. This resistance may be influenced by factors such as patient age, prior hospitalizations, and previous exposure to antibiotics ^34^^,^^39^^,^^40^. Notably, we observed elevated resistance rates to trimethoprim/sulfamethoxazole, consistent with findings reported in other countries ^37^^,^^38^^,^^41^. Given that local resistance to this drug combination exceeds the 20% threshold established by the *Ministerio de Salud, national* guidelines advise against its use for treating urinary tract infections ^42^. In contrast, nitrofurantoin has shown sustained efficacy against *E. coli*, as also reported in studies from multiple countries ^43^^-^^45^. These findings are crucial for guiding empirical antibiotic treatment of this type of infections in Colombia.

In line with previous studies ^45^^,^^46^, the *bla*_*TEM*_ gene family was more widespread across the isolates under study, despite some showing a greater prevalence of *bla*_*CTX*_ *_*_*M”*_ The significance of these two genes in uropathogenic *E. coli* has been generally recognized in recent studies ^47^^,^^48^.

A common approach in this context is the method suggested by Clermont *et al*. ^12^, which involves categorizing *E. coli* isolates into seven distinct phylogenetic clusters: A, B1, B2, C, D, E, and F, by amplifying portions of the *chuA, yjaA,* TspE4C2, and *arpA* genes using multiplex PCR. Several studies have demonstrated an association between specific phylogroups and the pathophysiology ^49^^,^^50^. For instance, most commensal bacteria are categorized in the phylogroups B1 or A, whereas isolates associated with extra-intestinal infections are in subgroup B2 ^12^^,^^51^. While this classification is helpful, each phylogroup has significant genetic variation. Therefore, it is essential to devise methods that complement or provide more specific phylogroup subtypes. Additional genotyping techniques like MLST ^10^^,^^16^ and WGS ^17^ should be performed to ascertain the characteristics of these isolates.

Extraintestinal infections, virulence factors, and biofilm formation highly correlate with the two prevailing phylogenetic groups, D and B2 ^52^^,^^53^. Previous studies have also correlated significantly phylogenetic group B2 and recurrent urinary tract infections ^54^. Thus, our findings offer novel insights into the attributes of the isolates identified in the region.

The CRISPR 2.1 *locus* polymorphism has been proposed as a useful auxiliary marker for bacterial genotyping, as it offers a simple yet informative method for differentiating *E. coli* isolates ^22^. In our study, length variations in this region provided valuable discrimination among isolates, supporting its contribution to molecular epidemiology. At the molecular level, this polymorphism arises from differences in the number and length of spacers-key structural components of the CRISPR/Cas system ^29^^,^^55^. In *E. coli*, this array typically contains 10-14 repeats but can range from 2 to 32 ^56^. Despite the observed polymorphism, our data revealed no significant association between the presence or absence of CRISPR 2.1 and the presence of resistance genes, a finding consistent with previous studies that found no such correlation in *E. coli*^57^^,^^58^.

Previous studies have shown that *E. coli* isolates belonging to phylogenetic group B2 generally lack the CRISPR 2.1 *locus*. It has been proposed that, over evolutionary time, the CRISPR-Cas system lost its immunological function in these strains due to the relatively low diversity and density of bacteriophages in their primary ecological niches ^56^^,^^59^. Interestingly, given that CRISPR-Cas systems function as barriers to the acquisition of foreign genetic material, including plasmids and transposons that often carry antibiotic resistance genes, one might expect an inverse relationship between the presence of CRISPR *loci* and the abundance of resistance determinants. However, in *E. coli*, the CRISPR system appears to have limited efficacy in restricting horizontal gene transfer compared to other bacterial species ^56^. In this sense, Touchon *et al.*^56^ found minimal sequence similarity between CRISPR spacers and plasmid sequences in various *E. coli* strains, suggesting a weakened immunological role. Therefore, the absence of CRISPR 2.1 in certain phylogenetic groups does not appear to confer an immunological disadvantage.

Our study contributes to the growing body of evidence supporting the use of CRISPR array polymorphism as a valuable marker for assessing genetic diversity among uropathogenic *E. coli* strains isolates. By confirming previously reported associations in a new geographic and clinical context, our findings expand the understanding of how CRISPR 2.1 variability reflects evolutionary divergence among uropathogenic *E. coli* strains. This is, to our knowledge, the first report documenting CRISPR polymorphism in uropathogenic *E. coli* in Colombia. Our data provides a relevant baseline for future molecular epidemiological studies in the region, particularly those aimed at tracing the emergence and spread of antibiotic-resistant and virulent clones.

## Figures and Tables

**Supplementary figure 1.**
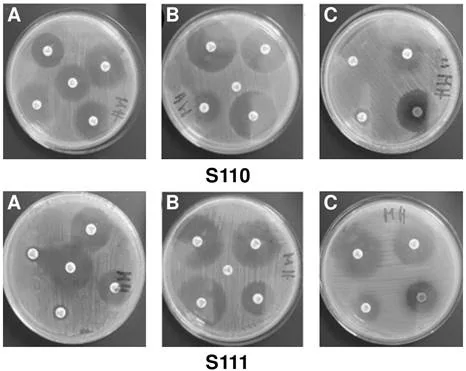
Representative antibiograms of uropathogenic *Escherichia coli* isolates S110 and Sill. Disk diffusion assays evaluated by the Kirby-Bauer disk diffusion method are shown for two representative isolates. The inhibition halos illustrate the antimicrobial susceptibility profile of each isolate.

A. Cefoxitin, cefotaxime, ceftriaxone, ceftazidime, piperacillin-tazobactam. B. Meropenem, imipenem, ertapenem, ampicillin, amikacin. C. Levofloxacin, nitrofurantoin, gentamicin, trimethoprim/sulfamethoxazole.
